# Experimental Cholelithiasis

**Published:** 1915-01

**Authors:** 


**Experimental Cholelithiasis.** Lameris, Zentralblatt für Chir., Feb. 24, 1914, considers Luschka's crypts as pathologic, occurring in chronic cholecystitis and at least tending to the formation of stones in the gall bladder itself. As their disappearance is not probable, he recommends cholecystectomy rather than cholecystotomy in cholelithiasis. Experimenting with rabbits he constricted the cystic duct and compressed the gall bladder to cause traumatism. 1 c.c. of 24-hour old typhoid culture was injected into the heart. Five of 24 animals died as the result of the operations. Nine developed calculi. Six of the remainder failed to do so, probably because the ligature about the systic duct caused complete occlusion. Luschka's crypts were found in various stages of development, containing epithelial debris and bile pigment, here and there already manifesting a concentric arrangement suggestive of subsequent development of calculi. No crypts were found in 13 normal rabbits.

